# Supplementary material for: Host age structure reshapes parasite symbiosis: collaboration begets pathogens, competition begets virulent mutualists
Source: Biol Direct. 2022 Nov 12;17:30. doi: 10.1186/s13062-022-00343-9 (PMC9652831; doi:10.1186/s13062-022-00343-9)
Supplement: Supplementary file 1 — Additional file 1. Fig. S1. Alternative model for collaboration among humans. A. Functional dependence on the population age structure for the fraction of younger hosts which reproduce under collaborative,$a=0, 0<b\le \frac{1}{2}$a=0,0<b≤12, and competitive,$b=0 , 0<a\le \frac{1}{2}$b=0,0<a≤12, conditions. Alternative functional form from that used in the main text. B. An example epidemic trajectory for $a=0.2$a=0.2 displaying the proportions of the three disease states. C. Epidemic trajectories for different degrees of collaboration with the younger, potentially reproductive, fraction of the population displayed. D. Population growth rate,$\frac{dN/dt}{N}$dN/dtN, during the epidemic phase (left) and relaxation into endemicity (right). E. Comparison of initial, disease free, growth rates and final growth rates at endemic equilibrium. F. Equivalent infection death rates for younger, potentially reproductive, hosts resulting in the same growth rate suppression for variable infection death rates among post-repro [file 13062_2022_343_MOESM1_ESM.pdf]

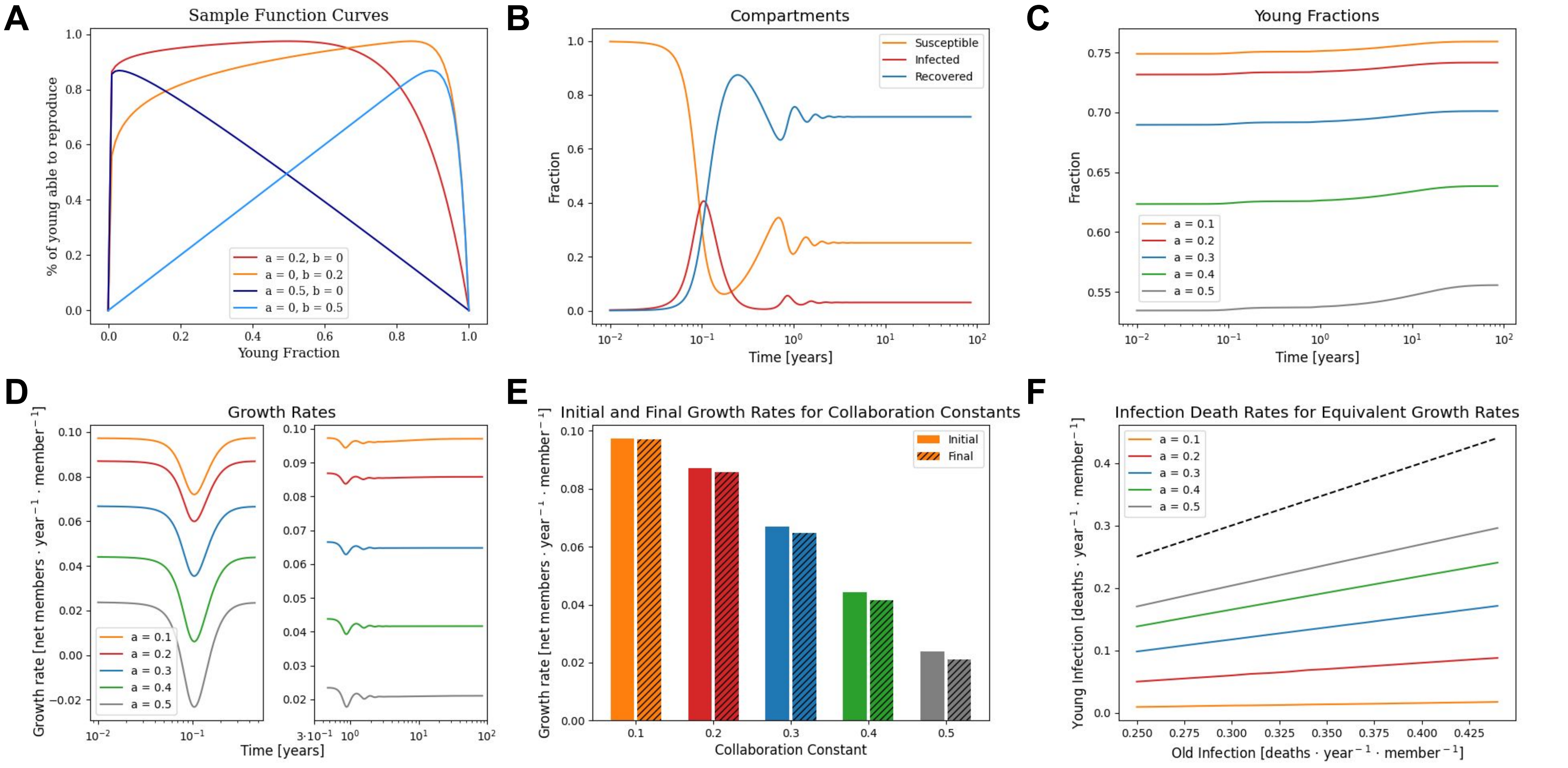

A
Sample Function Curves
% of young able to reproduce
Young Fraction
a = 0.2, b = 0
a = 0, b = 0.2
a = 0.5, b = 0
a = 0, b = 0.5
B
Compartments
Fraction
Time [years]
Susceptible
Infected
Recovered
C
Young Fractions
Fraction
Time [years]
a = 0.1
a = 0.2
a = 0.3
a = 0.4
a = 0.5
D
Growth Rates
Growth rate [net members · year⁻¹ · member⁻¹]
Time [years]
a = 0.1
a = 0.2
a = 0.3
a = 0.4
a = 0.5
E
Initial and Final Growth Rates for Collaboration Constants
Growth rate [net members · year⁻¹ · member⁻¹]
Collaboration Constant
Initial
Final
F
Infection Death Rates for Equivalent Growth Rates
Young Infection [deaths · year⁻¹ · member⁻¹]
Old Infection [deaths · year⁻¹ · member⁻¹]
a = 0.1
a = 0.2
a = 0.3
a = 0.4
a = 0.5

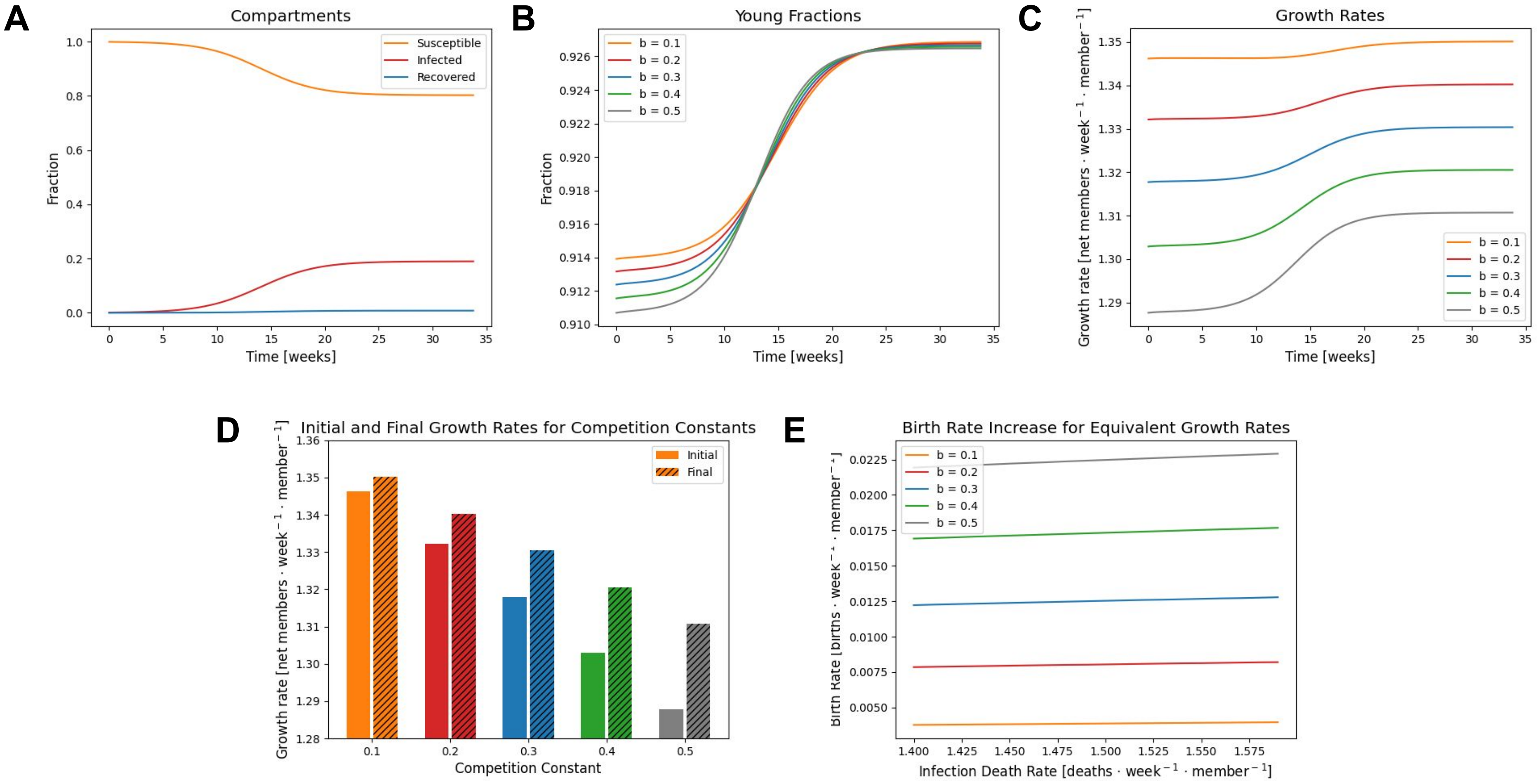
A
Compartments
Susceptible
Infected
Recovered
Fraction
Time [weeks]
B
Young Fractions
b = 0.1
b = 0.2
b = 0.3
b = 0.4
b = 0.5
Fraction
Time [weeks]
C
Growth Rates
Growth rate [net members · week^{-1} · member^{-1}]
b = 0.1
b = 0.2
b = 0.3
b = 0.4
b = 0.5
Time [weeks]
D
Initial and Final Growth Rates for Competition Constants
Initial
Final
Growth rate [net members · week^{-1} · member^{-1}]
Competition Constant
E
Birth Rate Increase for Equivalent Growth Rates
b = 0.1
b = 0.2
b = 0.3
b = 0.4
b = 0.5
Birth Rate [births · week^{-1} · member^{-1}]
Infection Death Rate [deaths · week^{-1} · member^{-1}]

Extended Data A: Specification of age structure

We begin with an age structured population, divided into two compartments of young, potentially reproductive members, Y, and older, post-reproductive members, O. The proportion of young hosts which are able to reproduce is goverend by the proportion of the population which is young:

$$f\left(\frac{Y}{Y+O}\right) = cos^{10a}\left(\frac{\pi}{2}\frac{Y}{Y+O}\right)cos^{10b}\left(\frac{\pi}{2}\frac{Y}{Y+O}\right)$$

unless otherwise specified, and:

$$f\left(\frac{Y}{Y+O}\right) = \left(1-\frac{Y}{Y+O}^{1000\left(\frac{1}{2}-a\right)}\right)\frac{Y}{Y+O}^{1000\left(b-\frac{1}{2}\right)}$$

for Supplementary Figure 1 where $a = 0$, $0 < b < \frac{1}{2}$ or $b = 0$, $0 < a < \frac{1}{2}$. Reproductive hosts give birth at rate k_B and age into the post-reproductive compartment at rate k_A. Post-reproductive hosts die at rate k_D. These dynamics are represented by the system of equations:

$$\begin{bmatrix} Y \\ O \end{bmatrix}' = \begin{bmatrix} f\left(\frac{Y}{Y+O}\right)k_B - k_A & 0 \\ k_A & -k_D \end{bmatrix}\begin{bmatrix} Y \\ O \end{bmatrix}$$

This system may be utilized to derive the steady state age distribution, defined by the ratio $\frac{Y}{Y+O}$:

$$0 = \left(\frac{Y}{Y+O}\right)' = \frac{Y'}{(Y+O)} - \frac{Y(Y+O)'}{(Y+O)^2} = \frac{Y}{Y+O}\left(f\left(\frac{Y}{Y+O}\right)k_B + k_D - k_A\right) - \left(\frac{Y}{Y+O}\right)^2\left(f\left(\frac{Y}{Y+O}\right)k_B + k_D\right)$$

which may be solved numerically for arbitrary $f\left(\frac{Y}{Y+O}\right)$.

Extended Data B: Construction of epidemiological compartment model

We may now introduce the age structured population defined in the previous section, initialized with the steady state age distribution, to a pathogen. Hosts may be susceptible (S_Y,S_O), infected and infectious (I_Y,I_O), or recovered and immune (R_Y,R_O). Susceptible young/older hosts are infected at rate $k_I\frac{I}{N}S_{Y/O}$ where $I \equiv I_Y + I_O$ and N is the sum over all compartments, the total population. The death rate of young and old infected hosts due to infection may differ, $k_{DYI/DOI}$, and all recovered hosts may return to a susceptible compartment at rate k_L due to a loss of immunity. These dynamics yield the system of equations:

$$d\begin{bmatrix} S_Y \\ S_O \\ I_Y \\ I_O \\ R_Y \\ R_O \end{bmatrix}/dt = \begin{bmatrix} f(\frac{Y}{N})k_B - \frac{k_I I}{N} - k_A & 0 & f(\frac{Y}{N})k_B & 0 & f(\frac{Y}{N})k_B + k_L & 0 \\ k_A & -\frac{k_I I}{N} - k_D & 0 & 0 & 0 & k_L \\ \frac{k_I I}{N} & 0 & -k_R - k_{DYI} - k_A & 0 & 0 & 0 \\ 0 & \frac{k_I I}{N} & k_A & -k_R - k_{DOI} - k_D & 0 & 0 \\ 0 & 0 & k_R & 0 & -k_L - k_A & 0 \\ 0 & 0 & 0 & k_R & k_A & -k_L - k_D \end{bmatrix}\begin{bmatrix} S_Y \\ S_O \\ I_Y \\ I_O \\ R_Y \\ R_O \end{bmatrix}$$

Each simulation is conducted as follows. Parameters a and b are selected and the equilibrium age distribution, $\frac{Y}{Y+O}$, as well as the growth rate, $(dN/dt)/N$ where N is the cumulative size of all compartments, are computed. Compartments are initialized to respect the equilibrium age distribution with infected compartments initialized to be 0.1% of the total population. The solution is then propagated using Python scipy method solve_ivp until a state of endemic equilibrium is reached where the measured rate of change of compartment proportions is negligible:

$$|\frac{d\frac{Y}{N}}{dt}|\frac{N}{Y} + |\frac{d\frac{O}{N}}{dt}|\frac{N}{O} + |\frac{d\frac{S}{N}}{dt}|\frac{N}{S} + |\frac{d\frac{I}{N}}{dt}|\frac{N}{I} + |\frac{d\frac{R}{N}}{dt}|\frac{N}{R} < 0.0001$$
